# Effects of Surface Charge and Functional Groups on the Adsorption and Binding Forms of Cu and Cd on Roots of *indica* and *japonica* Rice Cultivars

**DOI:** 10.3389/fpls.2017.01489

**Published:** 2017-08-24

**Authors:** Zhao-Dong Liu, Qin Zhou, Zhi-Neng Hong, Ren-Kou Xu

**Affiliations:** ^1^State Key Laboratory of Soil and Sustainable Agriculture, Institute of Soil Science, Chinese Academy of Sciences Nanjing, China; ^2^University of Chinese Academy of Sciences Beijing, China

**Keywords:** functional groups, heavy metal, *indica* rice, *japonica* rice, zeta potential

## Abstract

This work was designed to understand the mechanisms of adsorption of copper (Cu) and cadmium (Cd) on roots of *indica* and *japonica* varieties of rice. Six varieties each of *indica* and *japonica* rice were grown in hydroponics and the chemical properties of the root surface were analyzed, including surface charges and functional groups (-COO- groups) as measured by the streaming potential and attenuated total reflectance-Fourier transform infrared spectroscopy (ATR-FTIR). Binding forms of heavy metals adsorbed on rice roots were identified using sequential extraction methods. In rice roots exposed to Cu and Cd solutions, Cu existed mainly in both exchangeable and complexed forms, whereas Cd existed mainly in the exchangeable form. The amounts of exchangeable Cu and Cd and total adsorbed metal cations on the roots of *indica* varieties were significantly greater than those on the roots of *japonica* varieties, and the higher negative charges and the larger number of functional groups on the roots of *indica* varieties were responsible for their higher adsorption capacity and greater binding strength for Cu and Cd. Surface charge and functional groups on roots play an important role in the adsorption of Cu and Cd on the rice roots.

## Introduction

Rice is one of the most important crops in the world and the staple of choice in China: the average daily rice consumption per capita (219 g) in China is almost 50% more than the global average (148 g) (Hu et al., 2016). Rice needs irrigation, which makes paddy soils more prone than upland soils to being polluted by heavy metals, which, in turn, leads to accumulation of heavy metals in rice grains. In recent years, soils in the subtropical regions of southern China were found to be contaminated with cadmium (Cd) and copper (Cu), the major sources being mining and smelting (Liu et al., 2005; Li et al., 2014; Zhang et al., 2015). The health risk caused by heavy-metal-contaminated rice grains – particularly Cd contamination in regions and populations where rice is virtually the sole staple food – is a matter of public concern (Hu et al., 2016). Therefore, it is important to control the uptake and accumulation of Cd and Cu in rice grains to reduce the potential health risk to populations heavily dependent on rice (Sebastian and Prasad, 2014).

The amount of Cd accumulated and translocated in plants varies with species and with cultivars within species (Grant et al., 1998). Rice can be divided into two major sub-species, *japonica* and *indica* (Yoshihara et al., 2010). The potential for Cd accumulation in rice grains (Morishita et al., 1987; He et al., 2006; Uraguchi et al., 2009; Hseu et al., 2010; Arao and Ae, 2011), shoots (Uraguchi et al., 2009; Liu et al., 2010), and roots (Liu et al., 2010; Yoshihara et al., 2010) is higher in *indica* cultivars than in *japonica* cultivars. Much of the Cd taken up by plants is retained in roots, but a portion is translocated to the aerial portions of the plant and into the seed. Similar trends were observed for Cu and lead (Pb) accumulation in *indica* and *japonica* rice in China. When Cu concentrations in polished grains of 285 rice cultivars were measured, the average concentration in *indica* rice was found to be double of that in *japonica* rice (Yang et al., 1998). Similarly, Pb concentrations in rice grains of *indica* were also higher than those in *japonica* (Liu et al., 2011, 2013). However, the mechanism of accumulation explaining these differences remains unclear.

The root surface is the first barrier that anions and cations need to cross in order to enter the plant, especially in non-hyper accumulating species. Therefore, examining the adsorption of anions and cations on roots is the first step to a better understanding of the relationship between the concentrations of the anions and cations in soil solution and their contents within plants. Sorption of metals by roots is due to the binding and electrostatic attraction by a limited number of cation exchange and binding sites on the surface of the cells, which impart a negative charge to the root surfaces. The type and number of these sites vary with species because of the composition of root cells and cell wall constituents that carry the functional groups. Wang et al. (2015) used electrophoresis to measure the zeta potential of cell walls separated from rice roots and found that the zeta potential of Wuyunjing-7 (*japonica*), an aluminum-tolerant variety, was less negative than that of Yangdao-6 (*indica*). However, such differences in intact roots and their effects on the adsorption of heavy metals by the roots are yet to be studied.

Recently, a streaming potential intrument has been developed to measure the zeta potential of intact roots and was used for investigating the relationship between the zeta potential of rice roots and the adsorption of aluminium (Al) by those roots (Li et al., 2015; Liu et al., 2016). In the present experiment, we used the technique together with attenuated total reflectance-Fourier transform infrared spectroscopy (ATR-FTIR) (1) to ascertain whether the differences in root surface charges between *indica* and *japonica* are universal; (2) to examine the effects of these differences on the adsorption of Cu and Cd on rice roots; and (3) to find out the possible mechanisms of such adsorption.

## Materials and Methods

### Rice Culture

Twelve cultivars of rice (*Oryza sativa* L.) commonly grown in China were used, six each of *japonica* (HY1, WLJ1, WYJ7, LJ9, WYJ21, and NJ9108) and *indica* (YLY2, YD6, YLY800, LY808, SLY862, and LY1259). The rice seeds were surface-sterilized with 30% H_2_O_2_ for 15 min, washed thoroughly with deionized water, soaked in deionized water for 4 h, and germinated at 25°C in darkness on a piece of gauze placed inside a polythene container. When the seedlings had grown about 2 cm tall, they were moved to a growth chamber with day/night temperatures of 27/20°C, a day length of 14 h, a light intensity of 375 μmol photon m^-2^ s^-1^, and a relative humidity of 70% and grown in a modified nutrient solution recommended by the International Rice Research Institute without being aerated (Fan et al., 2007). The composition of the nutrient solution (all figures in millimoles) was as follows: 0.75 (NH_4_)_2_SO_4_, 1.5 NaNO_3_, 0.32 NaH_2_PO_4_⋅2H_2_O, 0.5 K_2_SO_4_, 1.7 MgSO_4_7H_2_O, and 1.0 CaCl_2_, supplemented with (all figures in micromoles) 9.1 MnCl_2_⋅4H_2_O, 0.16 CuSO_4_⋅5H_2_O, 0.15 ZnSO_4_⋅7H_2_O, 0.07 (NH_4_)_6_Mo_7_O_24_⋅4H_2_O, 18 H_3_BO_3_, and 40 FeSO_4_⋅7H_2_O-EDTA. The nutrient solution (pH 5.5) was renewed every 3 days. After 15 days, uniform-looking seedlings were selected and transplanted into PVC pots (14.5 cm tall and 10 cm in diameter, each holding 12 plants). The plants were carefully removed after 25 days and their roots, washed in deionized water, were used for the subsequent experiments.

### Separation of Binding Forms of Cu and Cd on Rice Roots

Before the adsorption experiments, the plants were placed in deionized water for 12 h to remove excess nutrient ions from the root surfaces. The roots, washed three times in deionized water and then carefully blotted, were immersed in 1 L solution of Cu or Cd, and magnetically stirred. There are three initial concentrations of 10, 40, and 100 μM for both metals. After 2 h, the roots were removed, washed twice in deionized water, blotted dry with filter paper, and placed sequentially in 1 L each of 0.1 M KNO_3_, 0.05 M EDTA-2Na (pH 6), and 0.01 M HCl, 1 h in each solution, to extract the exchangeable, complexed, and precipitated Cu or Cd from the root surfaces, respectively (Liu and Xu, 2015; Liu et al., 2016), and their contents in the extractants were determined using atomic absorption spectrophotometry (nov AA350, Analytik Jena AG, Jena, Germany). After the rice roots were exposed to metal solution for 2 h, the amount of Cu and Cd entered the symplasm during the experiment should be much lower, thus they were not measured and discussed in this study. Three replicates were maintained of each cultivar for each treatment.

The adsorption of Cu and Cd by rice roots was performed without oxygenation. It was possible that some of Cu(II) and Cd(II) were reduced to Cu(I) and Cd(I) by root exudates during the adsorption experiments. However, the amount of Cu(II) and Cd(II) reduced should be low due to weak reducing condition and short reaction time of 2 h.

### Measurement of Zeta Potential

The zeta potential of rice roots was measured using the streaming potential equipment developed by our group (Li et al., 2015). The roots were placed into the measuring cells (length, 3 cm; internal diameter, 1.4 cm) of the streaming potential equipment. A multimeter was used for measuring the streaming potential (ΔE, mV) through a pair of non-polarizable Ag/AgCl electrodes. A conductivity meter was used for measuring the electrical resistance of the measuring cell with a pair of platinum electrodes. All the electrodes were attached to the ends of the measuring cell. The difference in the pressure of liquid (ΔP, Pa) between the two ends of the measuring cell was recorded using a liquid manometer. Electrolyte solutions were pumped into the measuring cell, and the streaming potential was varied along a hydraulic gradient (ΔP, Pa), achieved by adjusting a valve, and the ratio of ΔE to ΔP was calculated. The zeta potential (ζ) was calculated using the Helmholtz–Smoluchowski equation (Childress and Elimelech, 1996) as follows:

$$\zeta =\frac{\Delta E}{\Delta P}\frac{\mu }{{\varepsilon \varepsilon }_{0}}\kappa$$

where ΔE is the streaming potential (mV); ΔP is the difference between the liquid pressures (Pa); μ is the dynamic viscosity of the solution (Pa S); ε is the permittivity of the test solution (F/m); ε_0_ is the permittivity of free space (F/m), and κ is the conductivity of the solution (S/m).

### ATR-FTIR Spectroscopic Analyses

The ATR-FTIR spectra of rice roots exposed to 100 μM Cu or 100 μM Cd were measured using a Nicolet iS10 FTIR spectrometer (Nicolet Analytical Instruments, Madison, WI, United States) equipped with an ATR diamond crystal (the roots were placed on the crystal). The spectral range was 650–4000 cm^-1^ with a resolution of 4 cm^-1^ and 64 scans.

### Data Analysis

The average values of Cu and Cd adsorbed by the roots of each of the 12 cultivars, six *japonica* and six *indica*, and their average zeta potentials were calculated and the results were expressed as means ± standard deviation. Data processing and statistical analyses were carried out using SPSS ver. 20.0 for Windows (Chicago, IL, United States). One-way analysis of variance (ANOVA) was used in all experiments to ascertain whether the differences between treatments were significant at *P* < 0.05 (Pearson’s correlation).

## Results and Discussion

### Distribution of Different Forms of Cu and Cd

**FIGURE 1:**
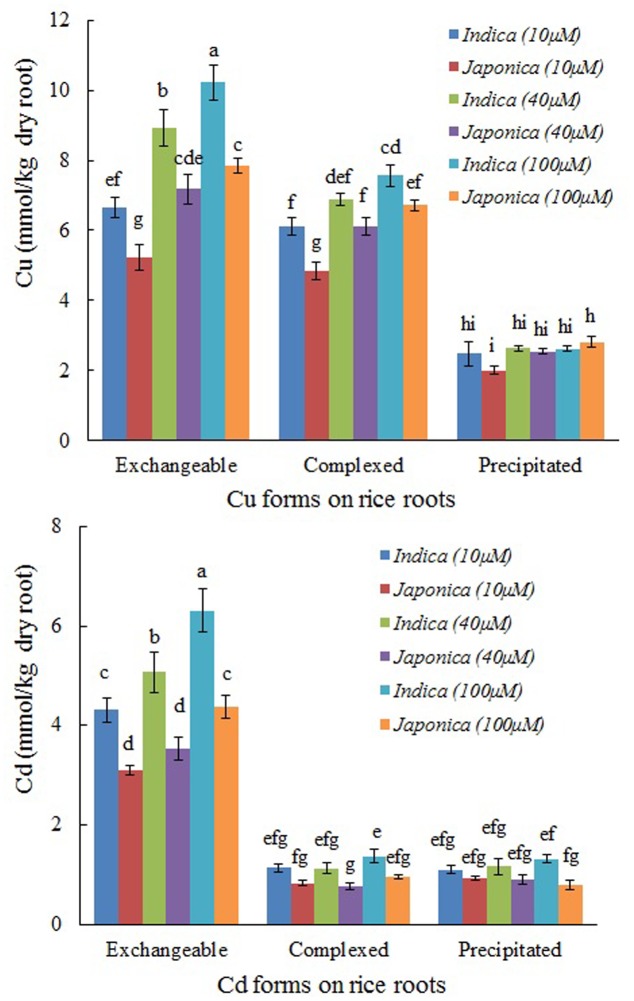
The concentrations of different binding forms of Cu and Cd on the roots of 40-day-old plants of *indica* and *japonica* varieties of rice. Values represent means from six *indica* and six *japonica* cultivars. Error bars are the standard errors of the means (*n* = 6). Different letters show significant differences among treatments and between *indica* and *japonica* (*P* < 0.05; LSD test).

After the roots had been exposed to Cu or Cd, the metal cations on the roots could be differentiated into their respective exchangeable, complexed, and precipitated forms (**Figure 1**). The exchangeable form of both metal cations accounted for the largest share, followed, in that order, by the complexed and the precipitated forms, the share of exchangeable Cd being greater than that of Cu. For example, when the concentration of metals was 100 μM, exchangeable Cu on the root surface accounted for 50.9% of the total Cu in *indica* and 48.7% in *japonica*, whereas the corresponding values for exchangeable Cd were 69.1 and 71.2%. However, complexed Cu accounted for a greater proportion in the total than complexed Cd did. At the initial concentration of 100 μM, complexed Cu contributed 37.0% of the total in *indica* and 38.7% in *japonica*, the corresponding figures for Cd being 15.2 and 15.6%. Exchangeable Cu and Cd on the roots of *indica* were significantly higher than those on the roots of *japonica* (*P* < 0.05) (**Figure 1**). The complexed forms of Cu and Cd were also higher in *indica* but the differences between the two types of rice were not significant except for complexed Cu at 100 μM (**Figure 1**). The differences between the two types of rice with respect to the precipitated forms of Cu and Cd were not significant.

### Zeta Potentials and Functional Groups on Roots of Different Rice Varieties

**FIGURE 2:**
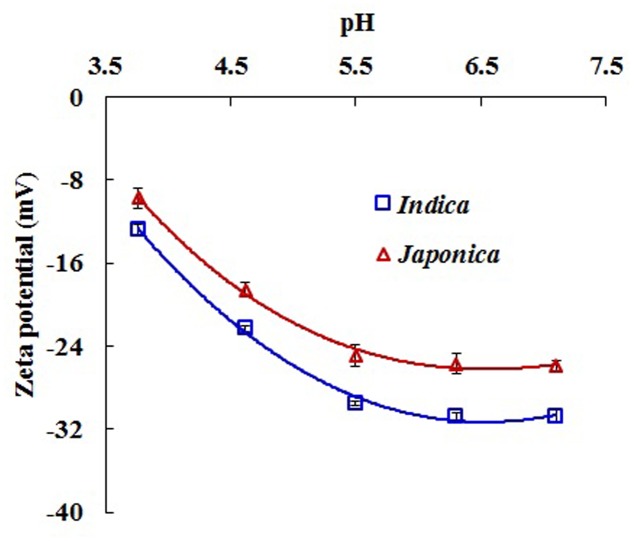
Zeta potentials of the roots of 40-day-old plants of *indica* and *japonica* varieties at different values of pH of the nutrient solution. Error bars are the standard errors of the means (*n* = 6).

The zeta potential of roots of 40-day-old plants of different rice varieties is shown in **Figure 2**. The means of zeta potential in **Figure 2** were calculated from six individual *indica* varieties or six individual *japonica* varieties (Supplementary Table S1). The zeta potential became more negative as the pH of the nutrient solution increased because of the increasing dissociation of functional groups on the roots, as was also observed earlier (Li et al., 2015; Liu et al., 2016), and was more negative in *indica* than in *japonica* (**Figure 2**): the difference was significant (*P* < 0.05), suggesting that root surfaces of *indica* rice carry a greater negative charge and thus have greater electrostatic attraction for metal cations.

**FIGURE 3:**
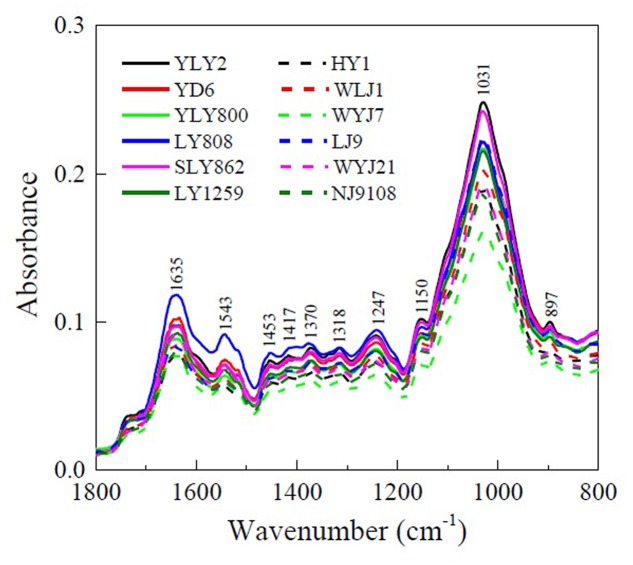
Attenuated total reflectance-Fourier transform infrared spectroscopy (ATR-FTIR) spectra of roots of six *indica* (YLY2, YD6, YLY800, LY808, SLY862, and LY1259) and six *japonica* (HY1, WLJ1, WYJ7, LJ9, WYJ21, and NJ9108) varieties.

The functional groups on rice roots are the major sources of the negative charge and binding sites for heavy metal cations. The ATR-FTIR spectra for the roots of *indica* and *japonica* varieties are shown in **Figure 3**. The absorption bands at 1635, 1543, and 1247 cm^-1^ were attributed to amide I (antisymmetric stretching vibrations of carboxyl), amide II (N–H bending vibrations), and amide III (C–N stretching and N–H bending vibrations) (Cai et al., 2014; Wang et al., 2015). The peak near 1417 cm^-1^ was assigned to symmetric -COO- stretching (Wang and Nielsen, 1992); the peaks at 1543, 1370, and 1318 cm^-1^ were assigned to the CH_2_ stretch of cellulose (Cai et al., 2014; Lv et al., 2016); and the 897 cm^-1^ peak was assigned to the β-anomeric bond of cellulose (Kačuráková et al., 2000; Zhong et al., 2011). The peaks at 1150 and 1031 cm^-1^ were assigned to C–O–C stretching or the skeletal vibration of lipids or cellulose and C–OH bending vibrations in carbohydrates, respectively (Kačuráková et al., 2000; Cai et al., 2014). No obvious differences in the locations of the absorption peaks were observed between the spectra of *indica* and *japonica*. However, the intensity of absorption peaks in the ATR-FTIR spectra of *indica* was higher than that of *japonica* (**Figure 3**). These results indicate that although both *indica* and *japonica* have the same surface functional groups on their roots, the groups are present in larger numbers on the roots of *indica*, which explains the greater negative charge on its roots.

### Effects of Surface Charge and Functional Groups on Binding Forms of Cu and Cd on Rice Roots

Rice varieties of the *japonica* group tolerate Al toxicity better than those of the *indica* group do (Ma et al., 2002; Watanabe and Okada, 2005; Yang et al., 2008; Famoso et al., 2010; Zhao et al., 2013). The varieties sensitive to Al carry a greater negative charge on their roots than those tolerant to Al (Yermiyahu et al., 1997; Wang et al., 2015), which is one of reasons for the lower tolerance of the sensitive varieties (Wang et al., 2015; Liu et al., 2016). In the present study, roots of six *indica* varieties were observed to carry a greater negative charge than that carried by roots of the six *japonica* varieties (**Figure 2**). When the roots were exposed to Cu or Cd, the exchangeable forms of adsorbed Cu and Cd on the roots of *indica* were significantly greater than those on the roots of *japonica* (**Figure 1**). The exchangeable forms of Cu and Cd were adsorbed on the negatively charged roots through electrostatic attraction between the heavy metal cations and the root surface. The greater negative charge on the roots of *indica* varieties adsorbed greater exchangeable Cu and Cd. The cations of Cu and Cd adsorbed electrostatically on rice roots were located in the diffuse layers of the electric double layers on the negatively charged surfaces of the roots. These cations are highly reactive and easily absorbed by plants. Therefore, the greater electrostatic adsorption of Cu and Cd on the roots of *indica* rice caused by the greater negative charge on the roots may be one of reasons for the higher uptake and accumulation of Cu and Cd by the *indica* group. Liu et al. (2010) discovered that Cd concentrations in root tissues and shoots of Shanyou 63 (*indica*) were higher than that of Wuyunjing7 (*japonica*). Yoshihara et al. (2010) also found that the Cu concentration in roots was higher in *indica* than in *japonica* when the rice was exposed to the 10 μM Cu.

Of its three binding forms, Cd existed mainly as the exchangeable form on rice roots; the proportions of the complexed and the precipitated forms of Cd were much lower (**Figure 1**). On the other hand, Cu existed mainly in exchangeable and complexed forms; the proportion of precipitated Cu was much lower (**Figure 1**). Therefore, the surface charge on rice roots plays an important role in the adsorption of both heavy metal cations, especially that of Cd.

**FIGURE 4:**
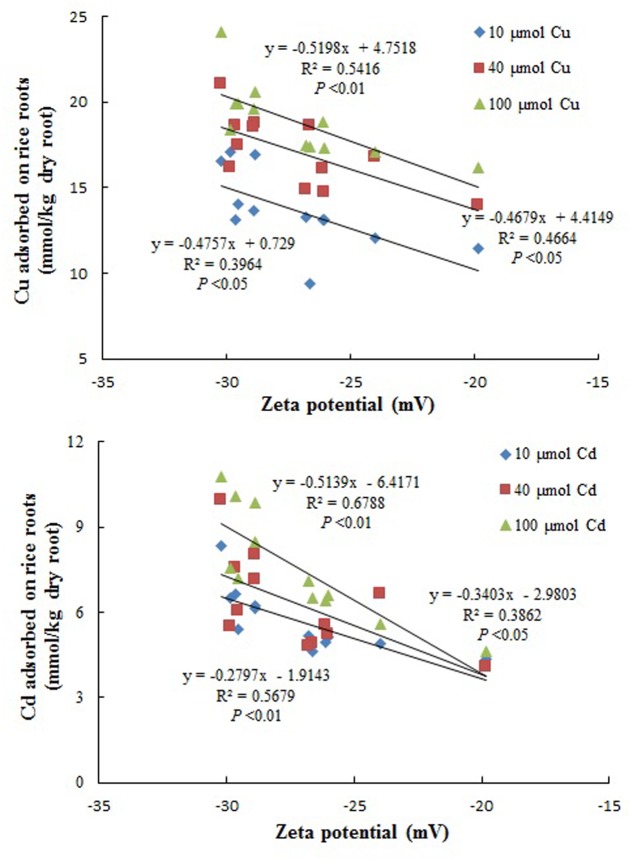
The relationships between the zeta potentials of the roots of the 12 rice cultivars tested in this study at pH5.5 and amounts of Cu and Cd adsorbed on the roots of these rice cultivars (Pearson’s correlation analysis).

Correlation analysis was performed between the zeta potential of the roots of each of the twelve rice cultivars tested in this study and amount of Cu and Cd adsorbed on the roots of these rice cultivars. Based on Pearson’s correlation analysis, there was a significant correlation between zeta potential of different rice roots at pH5.5 and amount of Cu and Cd adsorbed on the roots of these rice cultivars (**Figure 4**), which confirmed that surface charge was one of important factors determining the adsorption of Cu and Cd on the roots of different cultivars of rice. Compared with the *japonica* varieties, the *indica* varieties carried greater negative charge on their root surfaces, which may be one of the reasons why the *indica* varieties were more sensitive to Cu and Cd and adsorbed more Cu and Cd on their root surfaces than *japonica* varieties.

The functional groups on rice roots are major sources of the negative charge and of the binding sites for heavy metal cations. Plant roots are negatively charged owing to the deprotonation and protonation of the surface functional groups (-COOH, -OH, -NH_2_, and -H_2_PO_4_) on the cell walls and cell membranes of roots (Kinraide et al., 1992; Meychik et al., 2005; Wu and Hendershot, 2008). The intensity of absorption peaks in the ATR-FTIR spectra of the roots of *indica* was greater than that of the roots of *japonica* (**Figure 3**), suggesting that the functional groups were present in larger numbers on the roots of *indica* and were responsible for the greater negative charge and greater exchangeable Cu and Cd on its roots.

### Binding Strength of Cu and Cd

**FIGURE 5:**
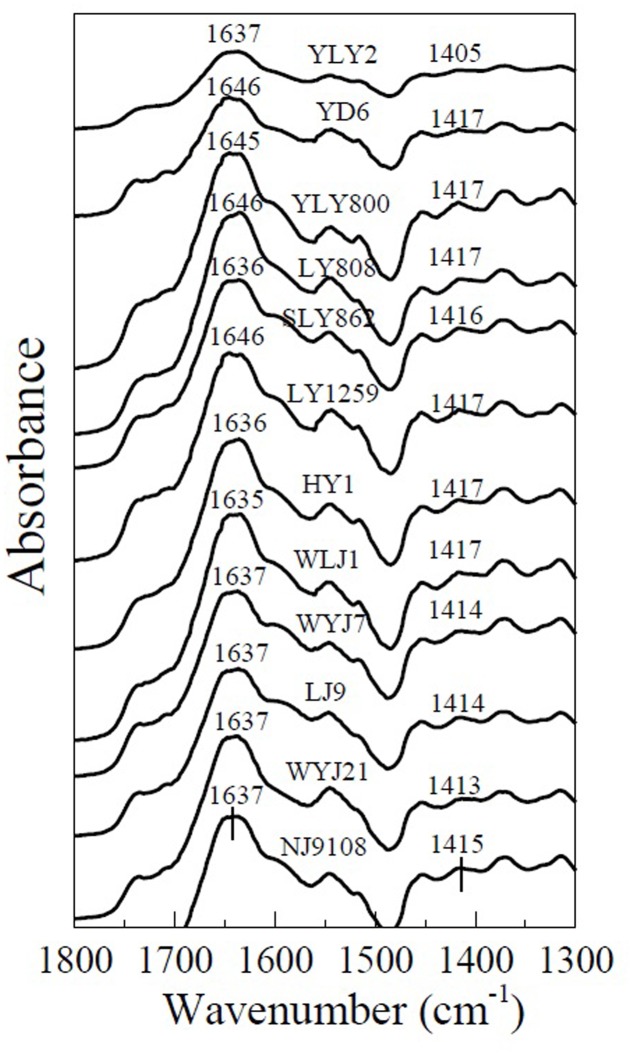
Attenuated total reflectance-Fourier transform infrared spectroscopy spectra of Cu-saturated roots of six *indica* (YLY2, YD6, YLY800, LY808, SLY862, and LY1259) and six *japonica* (HY1, WLJ1, WYJ7, LJ9, WYJ21, and NJ9108) varieties, showing the shift of antisymmetric (1637–1646 cm^-1^) and symmetric (1405–1417 cm^-1^) -COO- stretching due to Cu adsorption.

**FIGURE 6:**
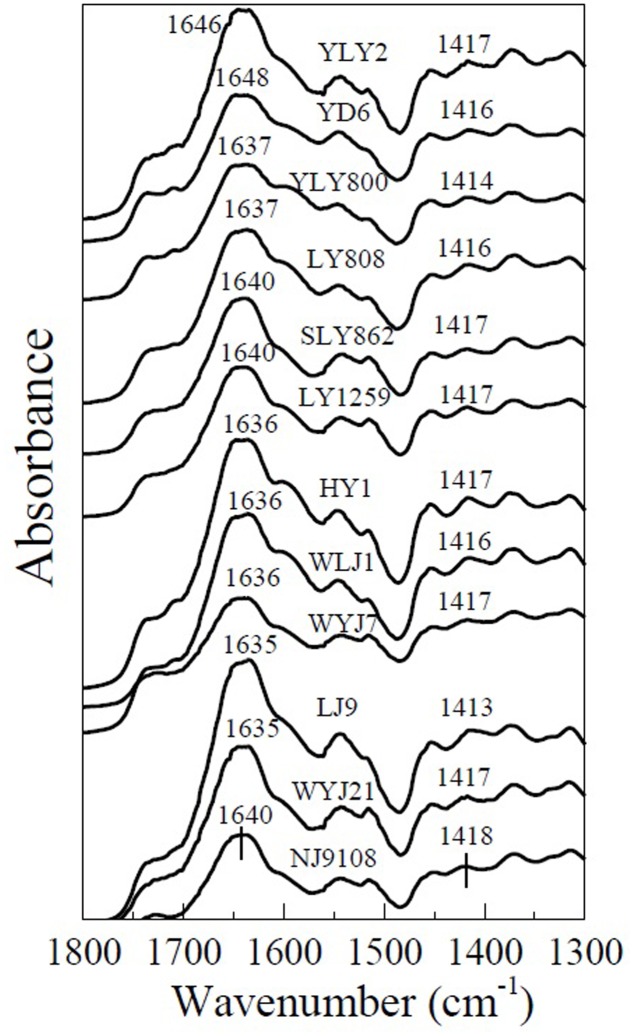
Attenuated total reflectance-Fourier transform infrared spectroscopy spectra of Cd-saturated roots of six *indica* (YLY2, YD6, YLY800, LY808, SLY862, and LY1259) and six *japonica* (HY1, WLJ1, WYJ7, LJ9, WYJ21, and NJ9108) varieties, showing the shift of antisymmetric (1637–1646 cm^-1^) and symmetric (1405–1417 cm^-1^) -COO- stretching due to Cd adsorption.

**FIGURE 7:**
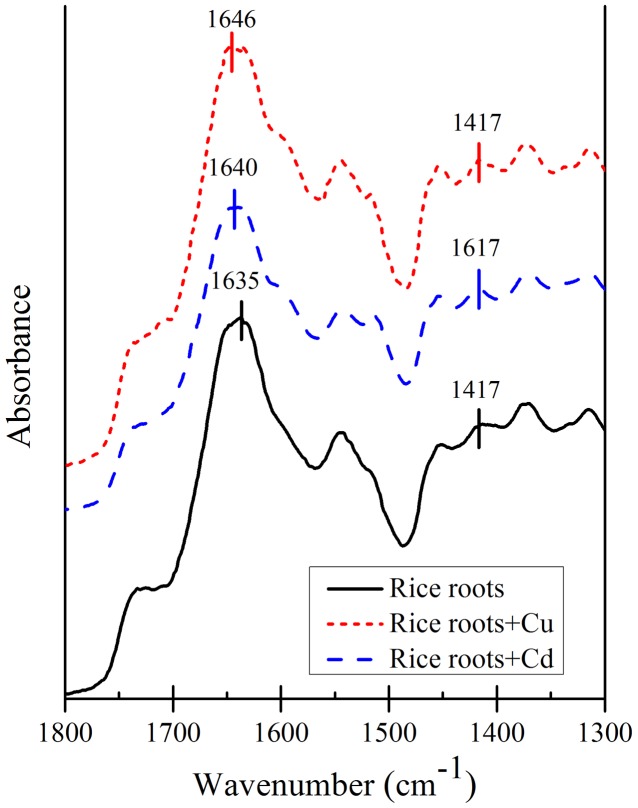
Attenuated total reflectance-Fourier transform infrared spectroscopy spectra of rice roots with and without Cu and Cd adsorbed (LY1259).

Wave number separation between the absorption peaks of antisymmetric and symmetric stretching vibrations has been used for determining the binding strength of carboxyl-containing organometallic complexes (Nakamoto, 1970). The spectra of the roots of different rice varieties with adsorbed Cu and Cd are shown in **Figures 5**, **6**. It is evident that adsorption of Cu and Cd changes the absorption peaks for antisymmetric and symmetric stretching vibrations of carboxyl. For example, the absorption peak of carboxyl on the rice roots of YL1259 at 1635 cm^-1^ was shifted to 1640 and 1646 cm^-1^ after adsorption of Cu and Cd on the rice roots (**Figure 7**). So were the directions of the shifts for the two types of stretching: the peak for the antisymmetric stretching vibration shifted to higher wave numbers as rice roots were saturated with Cu or Cd, whereas the peak for the symmetric stretching vibration shifted to lower wave numbers.

**FIGURE 8:**
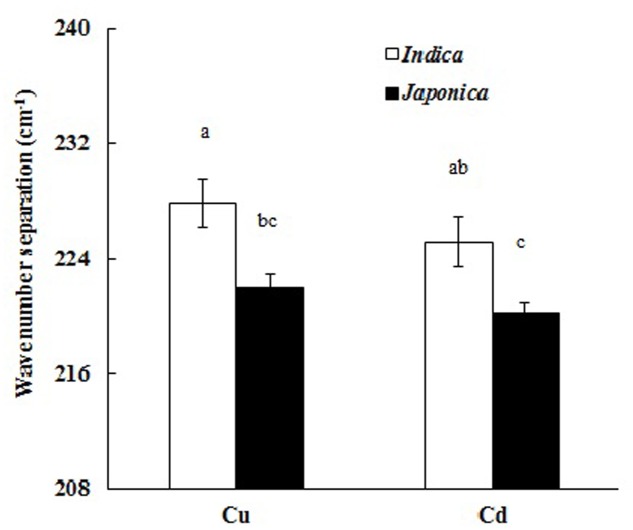
Comparison of wave number separations (difference between absorption peaks of antisymmetric and symmetric COO- stretching) due to adsorption of Cu or Cd by roots of *indica* and *japonica* varieties. Error bars are the standard errors of the means (*n* = 6). Different letters show significant differences among treatments (*P* < 0.05; LSD test).

The average wave number separation between the peaks of antisymmetric and symmetric stretching vibrations of carboxyl was calculated from the spectra in **Figure 5** (Cu) and **Figure 6** (Cd), and the results are shown in **Figure 8**. The data of the separations between the peaks of antisymmetric and symmetric stretching vibrations of carboxyl for six individual *indica* or *japonica* varieties were presented in Supplementary Table S2. After the roots were exposed to Cu or Cd, the wave number separation of *indica* between the peaks of antisymmetric and symmetric stretching vibrations was significantly greater than that of *japonica* (*P* < 0.05), suggesting that the strength of binding of Cu and Cd on roots of *indica* rice was greater than that of *japonica* rice. Therefore, the preference for Cu and Cd of the roots of *indica* was stronger than that of the roots of *japonica*. The results in **Figure 8** also indicate that the wave number separation of roots that had adsorbed Cu was greater than that of the roots that had adsorbed Cd, consistent with the greater amount of complexed Cu on rice roots than that of complexed Cd (**Figure 1**).

It is evident from the results shown in **Figure 2,** **3** that the roots of the two types differ in their surface chemical characteristics. Wave number separation between the absorption peaks of antisymmetric and symmetric stretching vibrations has been used for determining the binding strength of carboxyl-containing organometallic complexes (Nakamoto, 1970). Such a wave number separation of the two carboxyl bands can be explained as the result of an increasingly covalent metal-carboxyl bonding: the stronger the covalent bonding, the more asymmetric the structure of the carboxyl group. Any increase in the asymmetry of the carboxyl structure is reflected in greater separation of the two carboxyl vibrational bands (Nakamoto et al., 1961). Based on the above explanation, the bonding strength of Cu and Cd with the carboxyl groups on rice roots can be directly evaluated by comparing the wave number separations between the bands of antisymmetric and symmetric stretching vibrations. *Indica* varieties exhibited stronger interactions between their roots and Cu and Cd than *japonica* varieties did, as indicated by the greater wave number separation (**Figure 8**). This is to be expected, because *indica* varieties also carried a greater negative charge (**Figure 2**) and more adsorption sites for heavy metal cations (**Figure 3**) on their roots. Wang and Nielsen (1992) investigated the binding strength of Mn(II) to the cell walls of tobacco roots using wave number separation and found that the Mn-sensitive variety KY 14 exhibited a stronger interaction between the cell wall and the metal cation than the Mn-tolerant variety T.I. 1112 did, an observation consistent with the results reported in the present study.

The proportion of complexed Cu on rice roots was much greater than that of complexed Cd on the roots of the same type of rice (**Figure 1**). Soil organic matter and crop straw biochars behave similarly (Zhong et al., 2010; Xu and Zhao, 2013), because of the greater ability of organic functional groups to complex with Cu than with Cd (McBride et al., 1997). Nishizono et al. (1987) also found that the affinity of the cell wall of plant roots for Cu was markedly greater than that for Cd and Zn. These interpretations of the differences between the abilities of rice roots to form complexes with Cu and Cd are also supported by the results of wave number separations presented in **Figure 8**.

## Conclusion

Roots of *indica* rice carry a greater negative charge than those of *japonica* rice and have more functional groups on their surface. The differences in surface chemical properties of rice roots of *indica* and *japonica* were responsible for the differences in the amounts of Cu and Cd adsorbed by the roots of two types of rice: exchangeable Cu and Cd and total adsorbed Cu and Cd on the roots of *indica* varieties were significantly greater than these on the roots of *japonica* varieties (*P* < 0.05). The binding strength of the roots of *indica* varieties for Cu and Cd was greater than that of the roots of *japonica* varieties. When rice roots were exposed to Cu and Cd solutions under acidic conditions, Cu existed mainly in exchangeable and complexed forms on the roots, whereas Cd existed mainly in the exchangeable form. Therefore, the surface charge and the number of functional groups on rice roots play an important role in the adsorption of Cu and Cd by roots. The greater electrostatic adsorption of Cu and Cd by the roots of *indica* due to the greater negative charge on the roots may be one reason for the greater uptake and accumulation of Cu and Cd by *indica* rice as compared to *japonica* rice. These findings are of fundamental significance in understanding the mechanisms of the uptake and accumulation of Cu and Cd by *indica* and *japonica* varieties of rice and, consequently, the environmental risks posed by the accumulation of these heavy metals in the grains of the two types of rice.

## Author Contributions

Z-DL and R-KX designed the experiments. Z-DL and QZ performed the experiments. Z-DL drafted the manuscript. R-KX and Z-NH revised the manuscript.

## Conflict of Interest Statement

The authors declare that the research was conducted in the absence of any commercial or financial relationships that could be construed as a potential conflict of interest.
