# Supplementary material for: Effects of Surface Charge and Functional Groups on the Adsorption and Binding Forms of Cu and Cd on Roots of indica and japonica Rice Cultivars
Source: Front Plant Sci. 2017 Aug 24;8:1489. doi: 10.3389/fpls.2017.01489 (PMC5609544; doi:10.3389/fpls.2017.01489)
Supplement: Supplementary file 1 [file Table_1.DOCX]

Table S1 The zeta potentials of 40-day-old roots of six *indica* and six *japonica* cultivars at different pHs

| *Indica* varieties | pH | Zeta potential (mV) | | | *Japonica* varieties | pH | Zeta potential (mV) | | |
| --- | --- | --- | --- | --- | --- | --- | --- | --- | --- |
| YLY2 | 3.76 | -10.2 | -12.1 | -12.1 | HY1 | 3.76 | -9.6 | -9.4 | -10.9 |
|  | 4.62 | -20.6 | -20.6 | -22.7 |  | 4.62 | -21.0 | -20.9 | -20.7 |
|  | 5.5 | -28.7 | -28.9 | -29.0 |  | 5.5 | -26.0 | -26.0 | -27.9 |
|  | 6.3 | -29.2 | -34.1 | -29.2 |  | 6.3 | -26.9 | -26.9 | -26.8 |
|  | 7.1 | -28.4 | -28.4 | -30.6 |  | 7.1 | -26.9 | -26.9 | -26.8 |
| YD6 | 3.76 | -11.1 | -11.1 | -11.0 | WLJ1 | 3.76 | -8.4 | -10.0 | -9.9 |
|  | 4.62 | -22.7 | -22.6 | -22.4 |  | 4.62 | -17.8 | -19.6 | -19.5 |
|  | 5.5 | -29.6 | -29.5 | -29.5 |  | 5.5 | -24.6 | -26.8 | -26.7 |
|  | 6.3 | -29.4 | -32.0 | -31.9 |  | 6.3 | -25.1 | -26.9 | -27.1 |
|  | 7.1 | -31.9 | -31.9 | -31.9 |  | 7.1 | -24.4 | -26.4 | -26.4 |
| YLY800 | 3.76 | -13.5 | -15.7 | -10.6 | WYJ7 | 3.76 | -10.8 | -11.3 | -11.1 |
|  | 4.62 | -22.7 | -22.5 | -22.4 |  | 4.62 | -18.9 | -19.0 | -20.5 |
|  | 5.5 | -29.0 | -31.8 | -28.8 |  | 5.5 | -26.7 | -26.8 | -26.9 |
|  | 6.3 | -30.6 | -30.5 | -30.4 |  | 6.3 | -28.7 | -28.7 | -28.9 |
|  | 7.1 | -27.8 | -30.3 | -27.8 |  | 7.1 | -27.4 | -27.0 | -26.8 |
| LY808 | 3.76 | -13.3 | -13.1 | -13.0 | LJ9 | 3.76 | -5.3 | -5.2 | -5.1 |
|  | 4.62 | -23.6 | -23.4 | -20.5 |  | 4.62 | -14.5 | -16.3 | -16.3 |
|  | 5.5 | -28.9 | -29.0 | -28.8 |  | 5.5 | -27.0 | -26.9 | -24.5 |
|  | 6.3 | -28.3 | -31.0 | -31.0 |  | 6.3 | -23.5 | -25.4 | -25.5 |
|  | 7.1 | -35.0 | -30.0 | -29.9 |  | 7.1 | -23.6 | -25.7 | -25.7 |
| SLY862 | 3.76 | -14.0 | -13.8 | -13.6 | WYJ21 | 3.76 | -10.0 | -10.1 | -11.3 |
|  | 4.62 | -23.4 | -23.5 | -23.1 |  | 4.62 | -17.8 | -17.6 | -17.8 |
|  | 5.5 | -30.5 | -30.5 | -29.7 |  | 5.5 | -22.7 | -24.4 | -24.8 |
|  | 6.3 | -32.2 | -32.0 | -31.7 |  | 6.3 | -24.4 | -26.1 | -26.2 |
|  | 7.1 | -31.3 | -33.4 | -31.1 |  | 7.1 | -25.2 | -27.1 | -27.2 |
| LY1259 | 3.76 | -14.1 | -13.9 | -13.7 | NJ9108 | 3.76 | -12.7 | -12.5 | -12.4 |
|  | 4.62 | -21.0 | -20.8 | -23.2 |  | 4.62 | -16.4 | -21.4 | -18.7 |
|  | 5.5 | -26.0 | -31.5 | -31.4 |  | 5.5 | -21.0 | -19.3 | -19.2 |
|  | 6.3 | -29.3 | -31.3 | -29.6 |  | 6.3 | -20.9 | -23.4 | -20.7 |
|  | 7.1 | -30.9 | -31.3 | -31.3 |  | 7.1 | -23.9 | -24.3 | -24.3 |
