# Supplementary material for: Effects of Surface Charge and Functional Groups on the Adsorption and Binding Forms of Cu and Cd on Roots of indica and japonica Rice Cultivars
Source: Front Plant Sci. 2017 Aug 24;8:1489. doi: 10.3389/fpls.2017.01489 (PMC5609544; doi:10.3389/fpls.2017.01489)
Supplement: Supplementary file 2 [file Table_2.DOCX]

Table S2 Effect of Cu(II) and Cd(II) adsorption on wave number separations of 40-day-old roots of six *indica* and six *japonica* cultivars (differences between the absorption peaks of antisymmetric and symmetric COO- stretching)

| Rice varieties | | Cu(II) | | | Cd(II) | | |
| --- | --- | --- | --- | --- | --- | --- | --- |
|  |  | antisymmetric COO- stretching (cm^-1^) | symmetric COO- stretching (cm^-1^) | wave number separations (cm^-1^) | antisymmetric COO- stretching (cm^-1^) | symmetric COO- stretching (cm^-1^) | wave number separations (cm^-1^) |
| Indica | YLY2 | 1637 | 1405 | 232 | 1646 | 1417 | 229 |
|  | YD6 | 1646 | 1417 | 229 | 1648 | 1416 | 232 |
|  | YLY800 | 1645 | 1417 | 228 | 1637 | 1414 | 223 |
|  | LY808 | 1646 | 1417 | 229 | 1637 | 1416 | 221 |
|  | SLY862 | 1636 | 1416 | 220 | 1640 | 1417 | 223 |
|  | LY1259 | 1646 | 1417 | 229 | 1640 | 1417 | 223 |
| Japonica | HY1 | 1636 | 1417 | 219 | 1636 | 1417 | 219 |
|  | WLJ1 | 1635 | 1417 | 218 | 1636 | 1416 | 220 |
|  | WYJ7 | 1637 | 1414 | 223 | 1636 | 1417 | 219 |
|  | LJ9 | 1637 | 1414 | 223 | 1635 | 1413 | 222 |
|  | WYJ21 | 1637 | 1413 | 224 | 1635 | 1417 | 218 |
|  | NJ9108 | 1637 | 1415 | 222 | 1640 | 1418 | 222 |
